# Protective role of overweight in Alzheimer’s disease: New findings from Mendelian randomization study

**DOI:** 10.1002/alz.086859

**Published:** 2025-01-09

**Authors:** Hongzhe Duan, Rachel Holmes, Deqing Wu, Mary Feitosa, Anatoliy I Yashin, Konstantin Arbeev, Svetlana Ukraintseva

**Affiliations:** ^1^ Social Science Research Institute, Duke University, Durham, NC USA; ^2^ Washington University School of Medicine, St. Louis, MO USA

## Abstract

**Background:**

Weight loss has been associated with Alzheimer’s Disease (AD), suggesting the possibility that overweight may be protective against AD. Mendelian Randomization (MR) is a common method of revealing causal relationships in observational studies. Our recent MR analysis using the Health and Retirement Study (HRS) data found that overweight has a causal protective effect on late‐onset‐AD. Here we expand this strategy to two other cohorts, the Long Life Family Study (LLFS) and Framingham Heart Study (FHS), to examine causal role of overweight in AD in these data.

**Method:**

For this MR study, we created two binary variables: ‘exposure’ (group‐1 “overweight” BMI 25‐30 vs. group‐0 “normal weight” BMI 18.5‐25, at ages 65‐75) and ‘outcome’ (group‐1 “no AD” vs. group‐0 “AD onset after age 75”), using FHS original cohort (N = 610) and LLFS (N = 1979) data on individuals who survived age 75. To identify instrumental variables (*IVs*), we implemented a recently developed new strategy. We selected SNPs in eight obesity‐related genes (ADIPOQ, FTO, LEP, LEPR, INSIG2, MC4R, PCSK1, PPARG) and paired them with each other, then summed their minor alleles to create new ‘composite’ SNPs as candidate *IVs*. This strategy improved the MR effectiveness due to the exponentially increased number of to‐be‐tested *IVs*. R‐package *MendelianRandomization* was used. Analyses were stratified by sex. The relatedness of samples was addressed by using mixed model to calculate statistics. Causal effect was evaluated using weighted median and Inverse‐Variance Weighted (IVW). MR‐Egger regression was employed to address potential pleiotropy.

**Result:**

Being overweight at ages 65‐75 had significant causal protective effect on AD onset after age 75 in LLFS White females (N_IV = 61, weighted median causal estimate = 1.15, p‐value = 1.44E‐4; IVW causal estimate = 0.57, p‐value = 1.33E‐2) and FHS White males (N_IV = 12, weighted median causal estimate = 1.22, p‐value = 2.71E‐2; IVW causal estimate = 0.90, p‐value = 1.33E‐2). MR‐Egger regression intercepts in both cases were not distinct from the origin.

**Conclusion:**

Our MR study found causal protective effect of overweight on late‐onset‐AD in White participants of FHS and LLFS, in line with previous findings in HRS data. To confirm these findings in other race/ethnic groups, MR study of datasets with larger numbers of individuals of diverse backgrounds is warranted.

